# Artificial intelligence and tobacco use: A bibliometric analysis 1997–2026

**DOI:** 10.18332/tid/226603

**Published:** 2026-08-28

**Authors:** Shengbo Wang, Jiahao Zhang, Liushen Chen

**Affiliations:** 1 Department of Sociology, The Chinese University of Hong Kong, Hong Kong, China; 2 Southwestern University of Finance and Economics, Research Institute of Social Development. Chengdu, China

**Keywords:** artificial intelligence, tobacco, bibliometric analysis

## Abstract

**INTRODUCTION:**

Globally, tobacco use is a key public health issue. Exploring the impact of developing AI technologies on tobacco use and intervention will help promote the digital transformation of public health research. This study aims to map the trends, networks, and core themes of research at the intersection of artificial intelligence and tobacco use through visual analytics.

**METHODS:**

In this study, a bibliometric analysis was conducted on 335 articles on AI and tobacco use in the Web of Science core collection from 1997 to 2026. The search cutoff date was 8 April 2026. Descriptive and visual analysis were conducted on the publication trend, cooperation network, keyword co-occurrence, clustering, and burst of this literature.

**RESULTS:**

The speed of publication of literature in this field has increased significantly since 2018. In the cooperation network, national cooperation is dominated by the United States. Universities, rather than transnational institutions, significantly promote cooperation. The authors’ cooperation network is more dispersed. The characteristics of interdisciplinary cooperation are obvious. The research hotspots focus on several aspects. These include machine learning prediction of smoking, dialogue AI and robot interventions, and NLP and social media monitoring. Other topics encompass the analysis of smoking-cessation behavior among youth groups, combined with AI, deep learning, and behavioral analysis; and the evaluation of tobacco characteristics, tobacco products, media, and health. Keyword hotspots and bursts reveal that the field has recently paid special attention to the intervention of cutting-edge technologies in smoking and smoking cessation behavior.

**CONCLUSIONS:**

Through data mining and visualization technology, this study reveals the overall evolution, interaction mode, and key fields of AI and tobacco use knowledge. The results provide an important framework for further research.

## INTRODUCTION

Despite the declining trend in global tobacco use, the tobacco epidemic remains one of the most serious challenges in global public health in the 21st century^1^. The World Health Organization (WHO) estimates that tobacco use causes more than 8 million deaths worldwide each year and causes more than trillions of dollars in economic losses, including direct healthcare expenditures^2^. At the same time, the use of new tobacco products such as e-cigarettes is accelerating, and the use among youth groups is particularly prominent. There are tens of millions of young people aged 13–15 years around the world who are using e-cigarettes, and their average use rate is significantly higher than that of adults^3^. In the face of the changing tobacco epidemic situation and the increasing burden of disease, how to improve the accessibility, accuracy, and scalability of tobacco control interventions through technological innovation has become an important issue in the field of global tobacco control^4^. In 2025, the WHO Regional Committee for the Western Pacific (WPRO), in its policy agenda, explicitly listed ‘the application of artificial intelligence in healthcare’ and ‘strengthening tobacco control’ as the core issues for the future of regional health, and advocated the use of artificial intelligence and other technical means to promote equitable and technology-driven health systems^5^.

In recent years, the rapid evolution of artificial intelligence technology has provided a new analytical framework and technical path for analyzing the health damage effect of tobacco use behavior and optimizing tobacco control strategies^6^. Technical branches such as machine learning, natural language processing, and generative artificial intelligence have gradually penetrated multiple key links of tobacco control interventions, covering behavior prediction, content monitoring, and personalized service delivery^7^. From the perspective of the public health demand side, there is a significant gap between the huge global tobacco use population and the supply capacity of smoking cessation services. AI-driven intervention tools represented by chatbots, with their low cost, high scalability and all-weather accessibility, have shown great potential in bridging this gap between supply and demand^8^. From the perspective of technological development, the integration of AI and tobacco control also reflects the development trend of digital public health^9-11^.

Although the application of AI in tobacco control continues to expand, there are still key issues that need to be systematically addressed. Specifically, whether the current research hotspots match the public health needs, what is the evolution path between different research topics, and which directions are becoming emerging frontiers. Most of the existing reviews summarized the application and effect of AI in tobacco control from the content level^6,7,12^, but the systematic analysis of this interdisciplinary field from a bibliometric perspective remains limited. The bibliometric method can reveal the research structure and development trend through visualization, and has been widely used in the research of tobacco use and tobacco control^13^. However, the bibliometric analysis of this interdisciplinary field is still scarce. Therefore, applying this method to research on AI and tobacco use helps broaden the scholarly horizon and deepen our understanding of cutting-edge studies at the intersection of tobacco science and intelligent technologies.

Based on this, the study uses the bibliometric method and the Web of Science Core Collection database to systematically analyze the knowledge map of the research field on artificial intelligence and tobacco use. Specifically, this study aims to: 1) characterize the publication trends, core authors and institutions, major journals and high-impact literature in this field; 2) reveal research hotspots and knowledge structure through keyword co-occurrence and cluster analysis; and 3) combine burst detection and topic evolution analysis to identify potential frontier directions. The results will provide a systematic body of knowledge for researchers and policymakers in the field of tobacco control.

## METHODS

### Data source and search strategy

**Figure 1:**
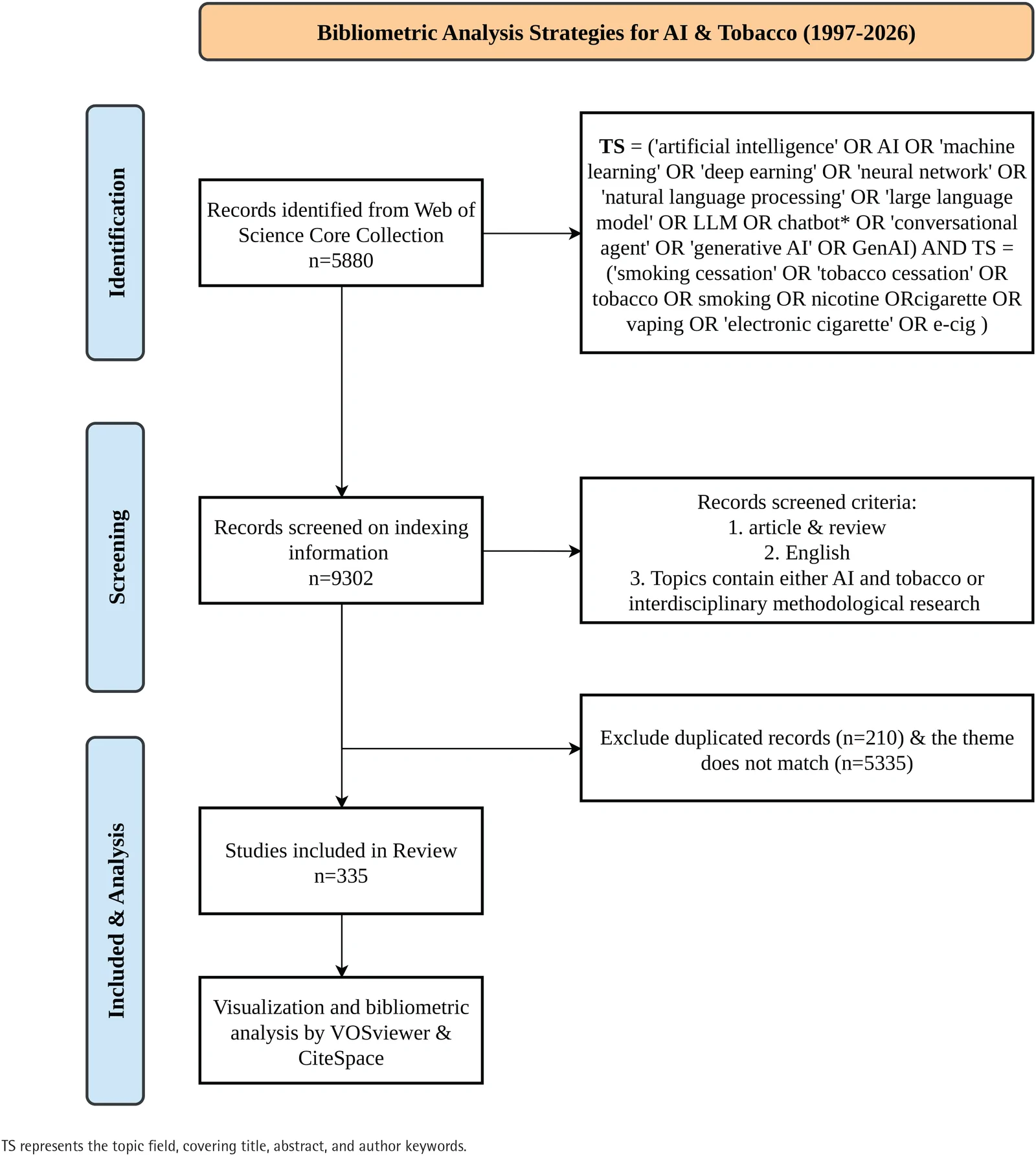
Flowchart of studies selection (1997–2026)

This study uses the Web of Science core collection as the data source. The retrieval strategy employs topic field retrieval, and the retrieval formula is constructed using the Boolean logic combination of the artificial intelligence term group and tobacco use term group. The topic retrieval formula is shown in Figure 1. TS represents the topic field, which covers the title, abstract, and author keywords. The types of literature are limited to articles and reviews, the language is limited to English, and the search date is 8 April 2026. The initial literature totaled 5880. The screening process was independently conducted by two authors, followed by cross-checking and discussion. A third author resolved discrepancies. Included studies had to meet the following criteria: 1) English-language research articles or reviews; 2) Studies explicitly integrating AI as content or methodology within tobacco-related topics; and 3) Substantive methodological research involving interdisciplinary approaches. Exclusion criteria comprised: 1) Non-English publications or non-article/review formats; 2) Studies mentioning only AI or tobacco use in isolation; 3) Studies using AI solely as a generic statistical tool or keyword without domain-specific application; and 4) Duplicate records. After removing 5545 irrelevant or duplicate records, the title information is exported in full-record and citation formats. Manual screening was based on the title, abstract, and keywords of the literature to determine whether it explicitly involved the application of artificial intelligence technology to tobacco use-related behavior or policy research. It excluded studies were those that only mentioned tobacco use or used AI only as a general statistical tool. Finally, a total of 335 articles were included in the analysis. The flowchart is shown in Figure 1.

### Data analysis methods

This study integrated bibliometric and knowledge map visualization methods^14,15^. First, descriptive statistical tools were used to analyze publication trends, core author productivity, and journal distribution. Second, VOSviewer 1.6.2 was used to generate author collaboration and keyword co-occurrence networks and to perform keyword cluster analysis. Third, CiteSpace 6.3.3 was used for burst term detection (threshold set at 0.3) and thematic evolution path analysis, identifying research frontiers and their dynamic evolution characteristics. In terms of metric calculation methods, Betweenness Centrality (BC) was computed using shortest-path algorithms to identify pivotal nodes that bridge different research domains. Network modularity (Q) was calculated using the Louvain algorithm to assess the clarity of thematic clusters.

## RESULTS

### Publication trends and journal distribution

**Figure 2:**
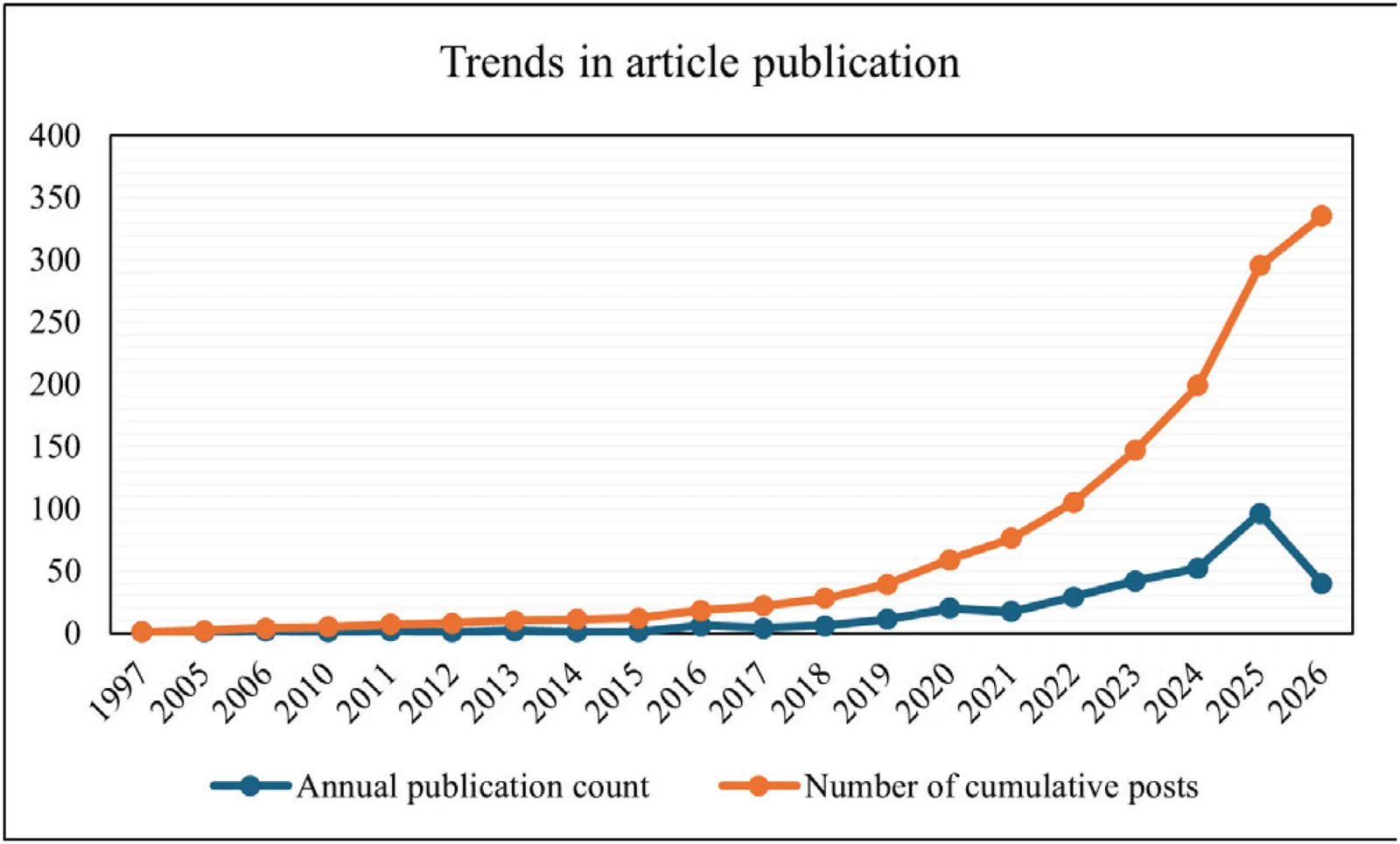
Publishing trend on AI and tobacco use. The blue line represents the annual number of publications related to AI and tobacco use from 1997 to 2026, while the orange line shows the cumulative publication count each year. The overall upward trend is evident

Figure 2 shows the temporal distribution of annual and cumulative publication counts in the field. The development of this interdisciplinary field can be divided into three main phases: before 2018, an embryonic period with low publication output (fewer than 10 articles per year); 2019–2022, a steady growth period with an average annual growth rate of 38.15%; and 2023 to the present, a rapid expansion period, with annual publications exceeding 50 and reaching a historical peak of 96.

The literature included in the analysis was published in 198 journals. Table 1 lists the top 10 journals in terms of the number of published articles. The top three journals are Journal of Medical Internet Research (18 articles), Nicotine & Tobacco Research (18 articles), and PLOS One (11 articles), which together account for about 14%. These are the three most concentrated journals in this field. Among the journals included in SSCI, Nicotine & Tobacco Research, Digital Health, and other journals in the field of social science and public health account for a relatively high proportion; in terms of SCI journals, Journal of Medical Internet Research, PLOS One, and other journals in the field of medical informatics and computer science have outstanding performance. The distribution of journals shows clear dual-track characteristics: research results are published in journals of public health and behavioral science, as well as in journals of computer science and medical informatics.

**Table 1:** Top 10 most prolific journals in AI and tobacco use research, 1997–2026

*Journal*	*Number*	*Proportion (%)*	*Type*	*IF*
Journal of Medical Internet Research	18	5.37	SCIE	6.0
Nicotine & Tobacco Research	18	5.37	SCIE/SSCI	3.0
PLOS One	11	3.28	SCIE	2.6
Digital Health	10	2.99	SCIE/SSCI	3.3
Drug and Alcohol Dependence	8	2.39	SCIE/SSCI	3.6
Scientific Reports	7	2.09	SCIE	3.9
Applied SciencesBasel	6	1.79	SCIE	2.5
BMC Public Health	6	1.79	SCIE	3.6
Substance Use & Misuse	5	1.49	SCIE/SSCI	1.7
Tobacco control	5	1.49	SCIE/SSCI	4.7

SCIE: Science Citation Index Expanded. SSCI: Social Sciences Citation Index. IF: Impact Factor.

### Collaboration networks: countries, institutions, authors, and disciplines

*Country collaboration network*

**Figure 3:**
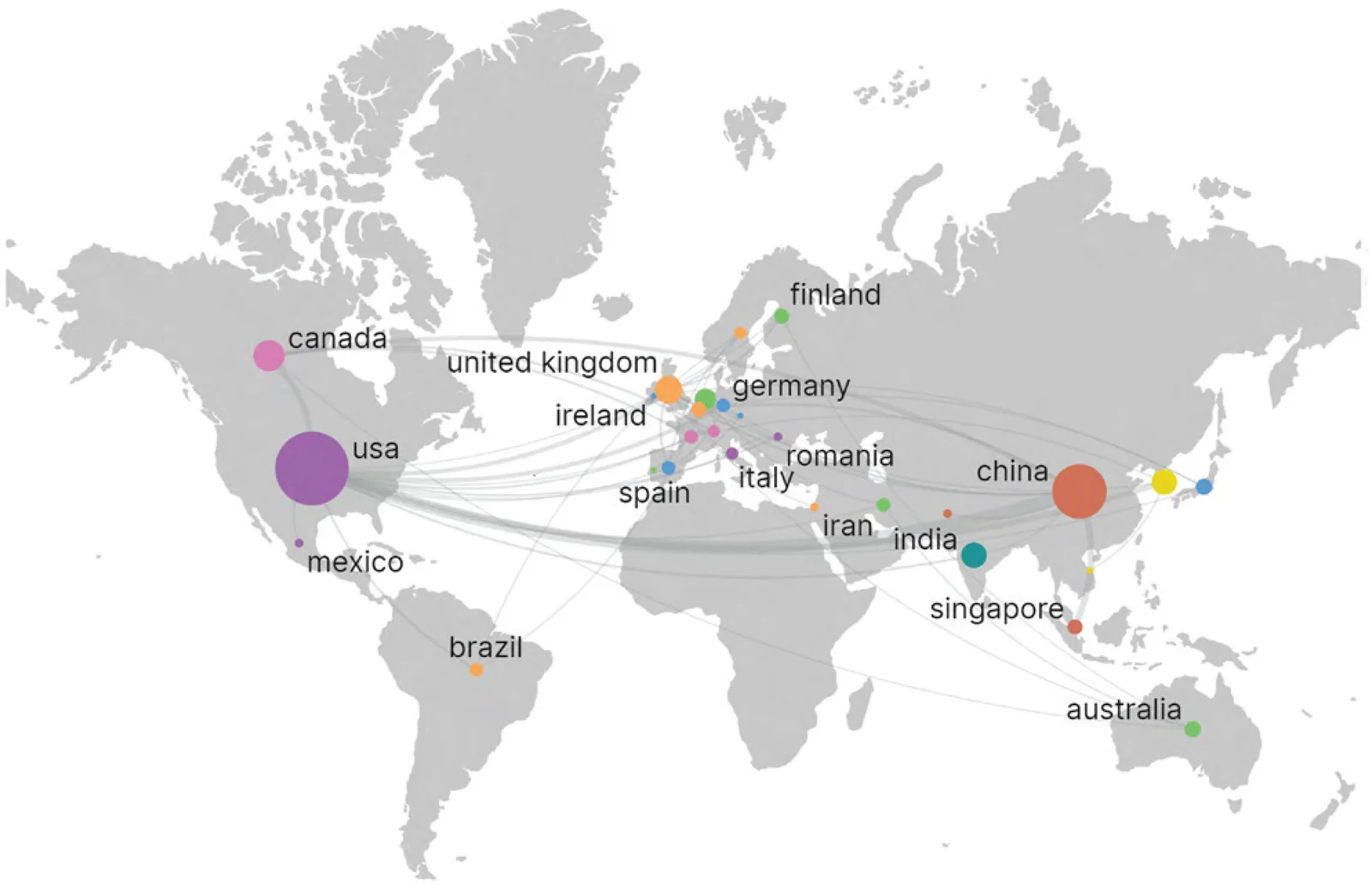
National-level Scientific Cooperation Network (1997–2026). Nodes represent countries and connecting lines indicate cooperation between them. The size of the nodes and the thickness of the connecting lines are proportional to the countries’ influence and the strength of their cooperation

The national-level scientific cooperation network is shown in Figure 3. The United States ranked first with 143 articles, accounting for 42.69% of the total literature, and dominated the field; China (78 articles, 23.28%), Canada (26 articles, 7.76%), the United Kingdom (20 articles, 5.97%), South Korea (17 articles, 5.07%) and India (17 articles, 5.07%) constituted the main research force. According to centrality indicators, the United States has the highest BC (0.38), followed by the United Kingdom (0.24) and China (0.13). The US–China, US–Canada, and US–UK cooperation networks are the main axes, but the overall density is low (0.062). High-income countries have an absolute advantage in the literature contribution in this field (about 83.28%), while the research participation of low- and middle-income countries is obviously insufficient^16^. The geographical distribution map of country cooperation (Figure 3) further shows that Europe and the United States have formed a relatively close cluster of cooperation. In contrast, the cooperation links among Asian, African, and Latin American countries are relatively sparse.

*Institution collaboration network*

**Figure 4:**
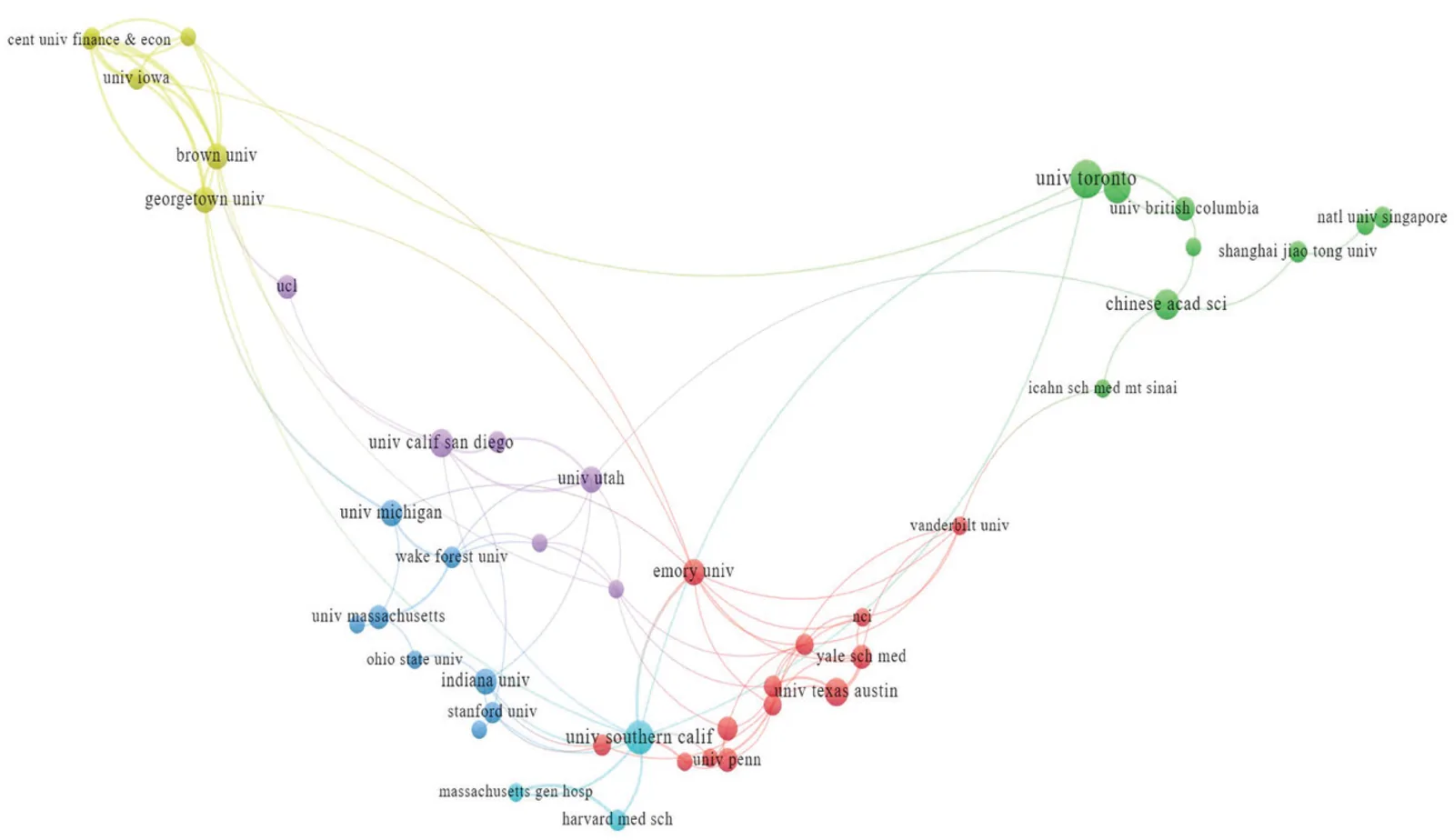
Institutional Collaboration Networks (1997–2026). Nodes represent institutions and connecting lines indicate collaborations between them. The size of the nodes is proportional to the journal’s influence. The thickness of the connecting lines is proportional to the strength of collaboration

The institutional-level cooperation network is shown in Figure 4. Among the top 10 research institutions, the University of California System (15 articles) and the China National Tobacco Corporation (12 articles) ranked first and second, respectively. University of Toronto, the Chinese Academy of Sciences, and the Center for Addiction & Mental Health-Canada ranked third (10 articles). However, the cluster analysis of institutional cooperation networks shows that cooperation mainly occurs between institutions within the same country or region, and the direct cooperation of transnational institutions is relatively limited. The network modularity index (Q=0.60) indicates that the institutional cooperation network is highly modular, forming a core institutional group with clear agglomeration. In addition, some high-yielding institutions are comprehensive universities and their medical schools, and professional tobacco control research institutions (such as national tobacco control centers) are not prominent in terms of centrality and publication volume in the network.

*Author collaboration network*

The results of the author collaboration network analysis are shown in Supplementary file Figure 1. The field has a stable but small core group of authors. According to Price’s Law, the minimum publication threshold for core authors is three articles. A total of 51 authors were identified as core authors, collectively contributing 187 articles (55.8% of the total), meeting the 50% standard expected by Price’s Law and indicating a stable core author group in the field. The top six high-output authors were P. Selby (7 articles); J. Rose (7 articles); N. Minian (5 articles); M. Ratto (5 articles); K. Mehra (4 articles); and L. Zawertailo (4 articles). The research topics of these authors are closely related, focusing on the application of chat robots and dialogic agents in smoking-cessation interventions. However, the overall density of the authors’ cooperation network is only 0.01.

### Interdisciplinary characteristics

Based on the Web of Science disciplinary classification system, the included documents involved 85 subject categories. Supplementary file Figure 2 (Disciplinary Collaboration Network) shows the distribution of publication volumes across disciplines. The top five disciplines by publication volume were: Public, Environmental & Occupational Health (73 articles, 21.79%); Health Care Sciences & Services (61 articles, 18.21%); Medical Informatics (60 articles, 17.91%); Substance Abuse (51 articles, 15.22%); and Psychiatry (29 articles, 8.66%). In terms of interdisciplinary degrees, approximately 59.4% of the documents involved two or more subject categories, with an average of 1.87 categories per document.

### Keyword co-occurrence analysis and research hotspots

*Keyword co-occurrence network*

Supplementary file Figure 3 shows the keyword co-occurrence network map, where node size represents keyword frequency, connections represent co-occurrence relationships, and node color represents cluster membership. The overall density of the network (the proportion of actual co-occurrence links to all possible keyword pairs) is 15.01%, and the total number of co-occurrence relationships is 2957. The top 20 high-frequency keywords are shown in Table 2. In addition to the core AI terms such as ‘machine learning’, ‘artificial intelligence’, ‘deep learning’, ‘chatbot’, and ‘natural language processing’. Tobacco-related terms such as ‘smoking’, ‘smoking cessation’, ‘tobacco’, ‘nicotine’, and ‘vaping’ also occupied a high frequency. It is worth noting that terms such as ‘United States’ (count=23), ‘health’ (count=23), ‘social media’ (count=22), and ‘cessation’ (count=22) appear more frequently.

**Table 2:** First 20 high-frequency keywords, 1997–2026

*Ranking*	*Keywords*	*Count*	*Ranking*	*Keywords*	*Count*
1	machine learning	102	11	social media	22
2	smoking	70	12	nicotine	20
3	smoking cessation	51	13	chatbot	19
4	tobacco	30	14	classification	19
5	artificial intelligence	28	15	natural language processing	18
6	smokers	23	16	cigarette-smoking	17
7	United states	23	17	vaping	16
8	health	23	18	nicotine dependence	15
9	deep learning	23	19	smoking-cessation	15
10	cessation	22	20	risk	15

*Cluster analysis and hotspot themes*

Supplementary file Figure 3 further presents the results of the keyword co-occurrence network clustering analysis, with a total of 7 main clusters identified. According to the results of cluster analysis and combined with high-frequency keywords, this field can be summarized into the following seven research hotspots:

1. Machine learning-driven smoking behavior prediction and risk stratification (green clustering). This hotspot occupies the core position of the keyword network, including keywords ‘machine learning’, ‘smoking’, ‘prediction’, ‘exposure’, ‘risk’, ‘cigarette-smoking’, etc.
2. Natural language processing and social media monitoring (purple clustering). The cluster contains keywords such as ‘natural language processing’, ‘social media’, ‘Twitter’, ‘tobacco’, ‘sentiment analysis’, ‘data-mining’, ‘impact’, etc.
3. Application of deep learning and computer vision in smoking behavior detection (golden clustering). The cluster contains ‘deep learning’, ‘convolutional neural network’, ‘classification’, ‘behavior’, etc.
4. A series of problems, such as e-cigarettes for adolescents and youth groups (blue clustering). The cluster contained keywords such as ‘vaping’, ‘adolescents’, ‘youth’, ‘depression’, and ‘cigarette use’.
5. Conversational AI, chatbots, and smoking cessation (red clustering). The cluster contained keywords such as ‘chatbot’, ‘conversational agent’, ‘smoking cessation’, ‘artificial intelligence’, and ‘smoking’.
6. Health, media, and tobacco products (orange cluster). The cluster contains keywords such as ‘health’, ‘media’, ‘advertising and promotion’, ‘tobacco industry’, and ‘tobacco’.
7. Feature prediction of nicotine dependence and tobacco use (cyan clustering). The cluster contains keywords such as ‘nicotine dependence’, ‘tobacco use’, ‘feature selection’, ‘predictive’, ‘models’, etc.

*Keyword density heatmap*

The keyword density heatmap is shown in Supplementary file Figure 4. The color in the figure changes from blue (low density) to green and then to yellow (high density), which intuitively presents the concentration of keywords in the research field. High-density areas were mainly clustered around words such as ‘machine learning’, ‘smoking’, ‘deep learning’, ‘smoking cessation’, ‘tobacco’, ‘health’, and ‘artificial intelligence’. Relatively speaking, the thermal strength of nodes such as ‘e-cigarettes’, ‘twitter’, and ‘mortality’ is relatively weak. It is worth noting that the heat of nodes such as ‘public health’, ‘large language model’, and ‘chatbot’ has shown a rapid upward trend in the near future^6,10^. The heatmap of superposition time coloring shows a transition from blue to green to yellow, reflecting the dynamic migration of the technology frontier.

*Keyword burst detection*

A total of 30 keywords with significant burst strength were identified. The top 15 keywords by burst strength are shown in Supplementary file Figure 5, with red segments indicating burst periods. In terms of burst strength, early burst terms (approx. 2006–2019) mainly included ‘data mining’ (intensity=1.67) and ‘support vector machines’ (intensity=1.68), reflecting the field’s early stage characterized by basic algorithm applications. Mid-period burst terms (approx. 2019–2022) included ‘cigarette smoking’ (intensity=3.55), ‘association’ (intensity=2.71), ‘text mining’ (intensity=2.37), ‘social media’ (intensity=1.74), and ‘natural language processing’ (intensity=1.67), indicating a gradual expansion from traditional machine learning algorithms to text mining, NLP, and other techniques. Recent burst terms (2022–present) with the strongest burst strength include ‘youth’ (intensity=2.02), ‘model’ (intensity=2.02), ‘conversational agent’ (intensity=1.91), and ‘smokers’ (intensity=1.84).

## DISCUSSION

### Publication trends and field development characteristics

The bibliometric analysis of this study shows that the literature output in the field of artificial intelligence and tobacco use research has evolved from slow accumulation to rapid expansion. Before 2018, the number of publications in this cross-cutting field was limited, and the topics were scattered. From 2019 to 2022, it entered a period of steady growth, and machine learning and natural language processing technologies began to systematically intervene in tobacco control issues. Since 2023, it has shown accelerating growth, and the introduction of generative AI and large language models has further expanded the scope of applications. This growth trajectory is consistent with the acceleration of global digital public health policy and the increasing emphasis on digital smoking cessation tools by the World Health Organization Framework Convention on Tobacco Control ( WHO FCTC ) in the time dimension^17,18^.

In terms of collaboration networks, at the country level, the United States dominates the field in terms of publication volume and centrality, with China, the United Kingdom, and Canada constituting major research forces. The collaboration network shows a bilateral and multilateral pattern primarily structured around US–China, US–UK, and US–Canada axes, but the depth and breadth of cross-regional collaboration still have substantial room for improvement. At the institutional level, research capacity is concentrated mainly in universities, with limited cross-border collaboration. The author collaboration network similarly shows fragmentation, with a limited high-output author group and no stable core–periphery structure yet formed. This finding addresses the gap in existing AI- and tobacco-related review studies regarding the neglect of collaborative relationships among research topics^6,19^. Notably, however, it diverges from broader tobacco research, which exhibits more frequent international collaboration and a distinctly global landscape^20^.

From a disciplinary perspective, the field exhibits significant interdisciplinary characteristics. Public, Environmental & Occupational Health; Health Care Sciences & Services; Medical Informatics; Substance Abuse; and Psychiatry constitute the five pillar disciplines, each corresponding to different research orientations. Public health focuses on evaluating the population health effects of AI interventions^21^. Medical informatics and computer science concentrate on building predictive models and decision support systems^22^; and behavioral science and psychiatry examine the mechanisms of AI in individual behavior change^9^. However, the participation of sociology, communication studies, and policy science remains relatively limited, with insufficient attention to the social-structural dimensions of tobacco use behavior and to policy translation pathways. This aligns closely with the current state of tobacco research^2,15^ and underscores the need for broader engagement and integration from the social sciences.

### Research hotspots and knowledge structure

Keywords in this field exhibit a high degree of correlation. Based on keyword co-occurrence networks and cluster analysis, we identified seven main research hotspots in the field, with a knowledge structure that exhibits a multi-layered progression from technical applications to public health practice. First, machine learning-driven prediction of smoking behavior and risk stratification constitutes the most intensive current research direction. Researchers widely use supervised learning algorithms such as Random Forest (RF), XGBoost, and Support Vector Machines (SVM) to predict smoking initiation, associated disease and mortality risks, and cessation success probabilities based on demographic characteristics, behavioral logs, and biomarker data^6,23^. Second, natural language processing is extensively applied to monitor tobacco content on social media, using pre-trained language models such as BERT and Transformer for data and text mining to identify and analyze smoking behavior, its control, and health communication content^24,25^. Third, personalized cessation interventions and message tailoring represent core applications of machine learning in behavioral support, with supervised learning dominating this subfield, covering message content selection, intervention timing optimization, and user feedback-driven adaptive adjustment^26^. Fourth, image recognition and sequence modeling techniques based on deep learning architectures such as CNNs and RNNs are used for smoker behavior detection, health status assessment, and disease risk identification^27,28^. Fifth, large language model-driven conversational intervention tools have become one of the most dynamic research directions, with LLM chatbots demonstrating strong effectiveness and high user acceptance in smoking cessation assistance^19,29^. Sixth, explainable AI and health equity focus on model decision transparency, the impact of algorithmic bias on health equity, and data privacy protection; the application of tools such as SHAP and LIME is still in its early stages^30^. Seventh, AI techniques are used to predict, understand, and intervene in smoking behaviors and attitudes among special populations such as adolescents and young adults^31,32^. The topics not only encompass the current mainstream research directions at the intersection of AI and tobacco studies^9,28^ but also bridge traditional technological approaches, such as SMS-based intervention program development^13^, with tobacco research.

### Research frontiers and future directions

Burst detection and thematic evolution analysis reveal three main frontier directions. First, large language models and multimodal deep learning are driving a paradigm shift in smoking cessation interventions from ‘reactive response’ to ‘real-time responsiveness’. Second, generative AI offers significant persuasive advantages in health communication, but it also carries the risk of misuse by the tobacco industry for marketing and promotion. This dual nature makes it an emerging governance issue that urgently requires attention. Third, the disciplinary gaps observed are also reflected in the burst term analysis, indicating that AI research remains largely confined to individual-level behavior, with insufficient attention to socioeconomic structural factors^6,33^.

These frontier trends also reflect several key shortcomings in current research. Specifically, the limited diversity of data in current studies restricts the external validity of models. Second, weak interdisciplinary integration means that social determinants are not adequately incorporated. Third, the lack of interpretability and an ethical governance framework restricts practical application, while the systematic path from evidence to policy transformation under the FCTC framework remains lacking. Future research should focus on building a global open data platform, deepening the integration of social and behavioral science, promoting the development of interpretable AI applications, and establishing technical evaluation standards and ethical review mechanisms under the FCTC framework. The use of AI technology in the tobacco industry poses a new challenge to the implementation of FCTC Article 5.3, and preventing the industry from using AI to erode public health policies should be a priority research topic.

### Limitations

There are some limitations in this study. Firstly, the data source is limited to the Web of Science core collection, and other related databases such as Scopus and PubMed are not included, which may omit some literature in the fields of computer science and regional public health. Secondly, the bibliometric indicators mainly reflect the scale of research output and the characteristics of the network structure, and have limited evaluative power for the scientific contribution and policy influence of individual research. Finally, as only English-language literature was included in the screening process, the findings of this study may be subject to language bias, overlooking relevant knowledge production from non-English-speaking regions.

## CONCLUSIONS

This study uses bibliometric and knowledge map analysis methods to systematically reveal the knowledge map and research patterns at the intersection of artificial intelligence and tobacco use. The study found that the number of publications in this field has continued to grow since 2018, forming a multi-research hotspot represented by machine learning prediction, social media content analysis, and conversational AI intervention. In general, interdisciplinary research at the intersection of artificial intelligence and tobacco control is in a period of rapid growth driven by technology. However, if the frontier algorithms are to be translated into practical public health outputs for tobacco control, it is still necessary to bridge multiple gaps across disciplinary boundaries, data bias, and policy transformation.

## Supplementary Material



## Data Availability

The data supporting this research are available from the authors on reasonable request.
