# Supplementary material for: Artificial intelligence and tobacco use: A bibliometric analysis 1997–2026
Source: Tob Induc Dis. 2026 Aug 28;24:10.18332/tid/226603. doi: 10.18332/tid/226603 (PMC13524066; doi:10.18332/tid/226603)

TID
Tobacco Induced Diseases

Supplementary file

© 2026 Wang S. et al.

DOI:
10.18332/tid/226603

The content has been provided by the author(s) and has not been reviewed, verified, or endorsed by European Publishing. It may not have undergone peer review. The views, opinions, and recommendations expressed are solely those of the author(s) and do not necessarily reflect the position of European Publishing. European Publishing accepts no responsibility or liability for any consequences arising from the use of, or reliance on, this content.

Figure 1. Author Collaboration Networks (1997-2026). Nodes represent authors and connecting lines indicate collaborations between them. The size of the nodes is proportional to authors' disciplines' influence. The thickness of the connecting lines is proportional to the strength of collaboration

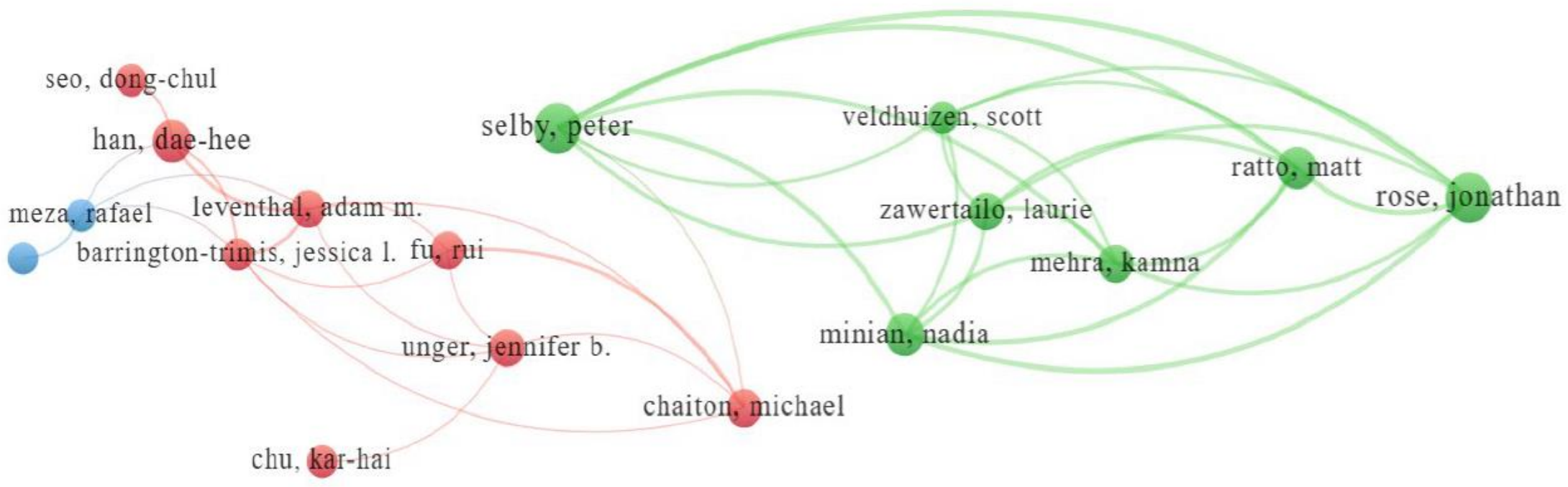

Figure 2. Disciplinary Collaboration Networks (1997-2026). Nodes represent disciplines and connecting lines indicate collaborations between them. The size of the nodes is proportional to the disciplines' influence. The thickness of the connecting lines is proportional to the strength of collaboration

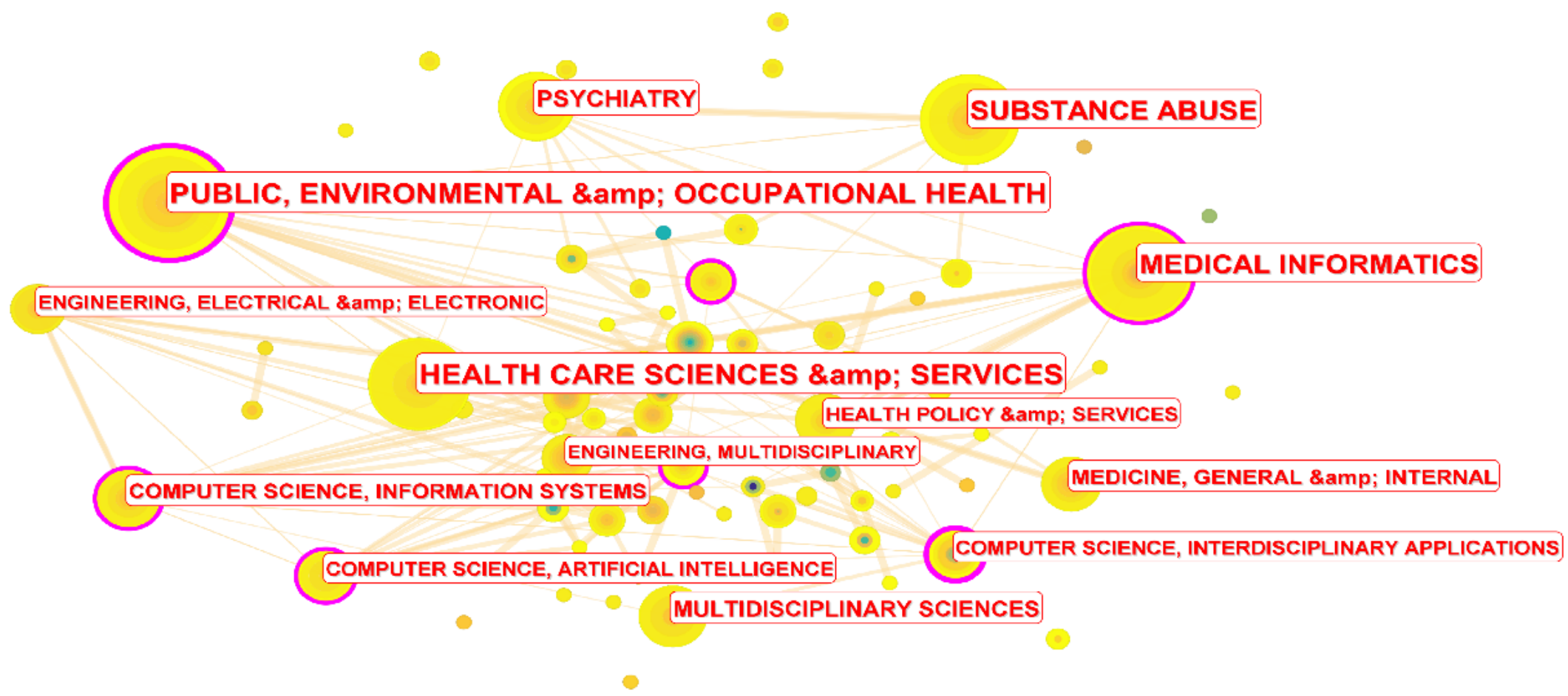

Figure 3. Keyword Co-occurrence Network (1997-2026). Nodes represent keywords and connecting lines indicate that the same keyword appears in different publications. The figure displays seven clusters

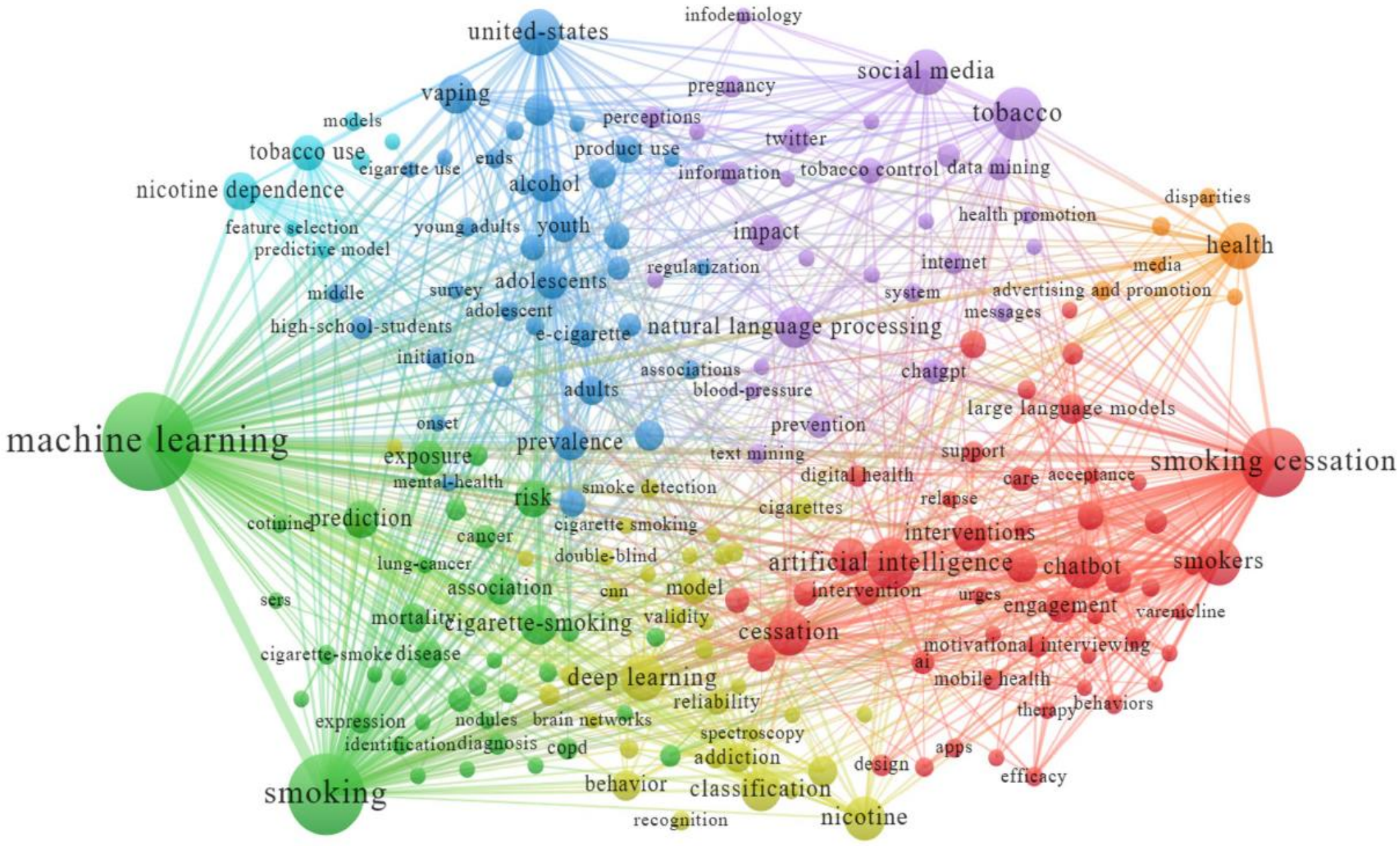

Figure 4. Density Heatmap (1997-2026). The color gradient from blue to green to yellow reflects the concentration of keywords within the research field

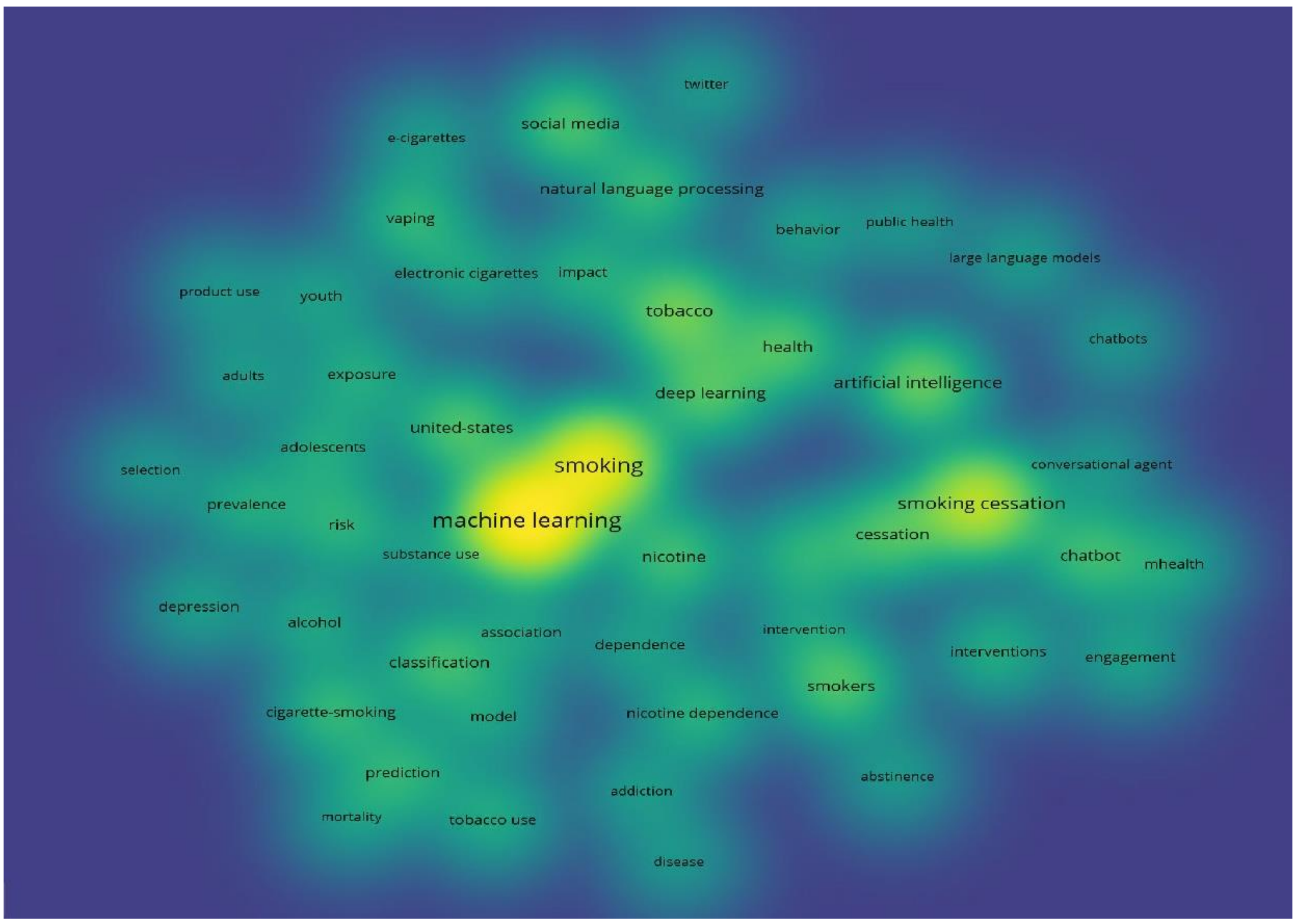

Figure 5. Bursts (1997-2026). Blue line segments mark the start and end years of publication for articles, while red line segments highlight periods of a sharp increase in citation counts

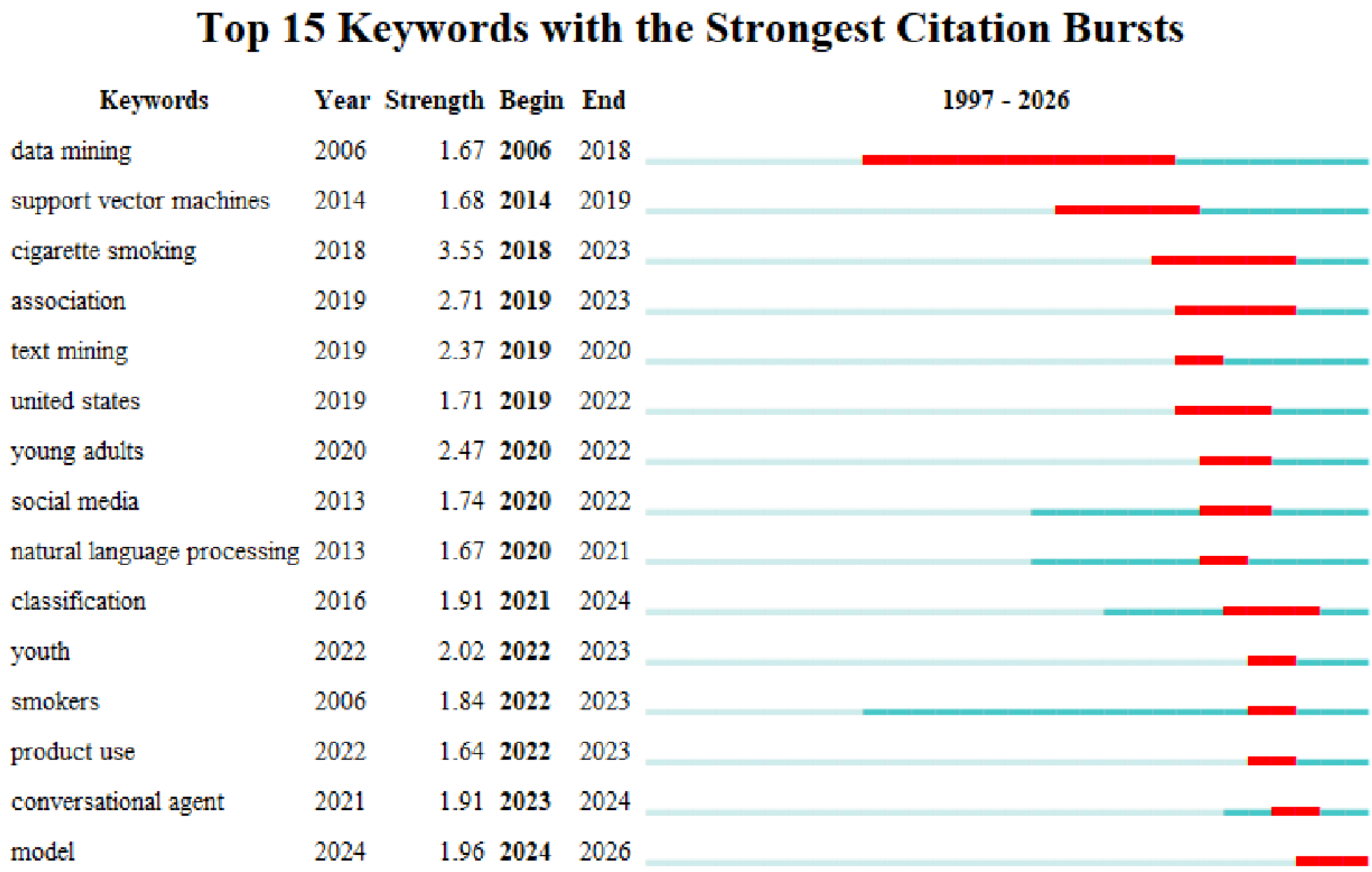

Supplement: Supplementary file 1 [file TID-24-151-s1.pdf]
